# Trehalose as an osmolyte in *Candidatus* Accumulibacter phosphatis

**DOI:** 10.1007/s00253-020-10947-8

**Published:** 2020-10-19

**Authors:** Danny R. de Graaff, Mark C. M. van Loosdrecht, Mario Pronk

**Affiliations:** 1 Department of Biotechnology, Delft University of Technology, van der Maasweg 9, 2629 HZ Delft, The Netherlands; 2 Royal HaskoningDHV, Laan1914 35, 3800 AL Amersfoort, The Netherlands

**Keywords:** Accumulibacter, Trehalose, Phosphate, Seawater, Salinity, EBPR

## Abstract

**Abstract:**

*Candidatus* Accumulibacter phosphatis is an important microorganism for enhanced biological phosphorus removal (EBPR). In a previous study, we found a remarkable flexibility regarding salinity, since this same microorganism could thrive in both freshwater- and seawater-based environments, but the mechanism for the tolerance to saline conditions remained unknown. Here, we identified and described the role of trehalose as an osmolyte in *Ca.* Accumulibacter phosphatis. A freshwater-adapted culture was exposed to a single batch cycle of hyperosmotic and hypo-osmotic shock, which led to the release of trehalose up to 5.34 mg trehalose/g volatile suspended solids (VSS). Long-term adaptation to 30% seawater-based medium in a sequencing batch reactor (SBR) gave a stable operation with complete anaerobic uptake of acetate and propionate along with phosphate release of 0.73 Pmol/Cmol, and complete aerobic uptake of phosphate. Microbial analysis showed *Ca.* Accumulibacter phosphatis clade I as the dominant organism in both the freshwater- and seawater-adapted cultures (> 90% presence). Exposure of the seawater-adapted culture to a single batch cycle of hyperosmotic incubation and hypo-osmotic shock led to an increase in trehalose release upon hypo-osmotic shock when higher salinity is used for the hyperosmotic incubation. Maximum trehalose release upon hypo-osmotic shock was achieved after hyperosmotic incubation with 3× salinity increase relative to the salinity in the SBR adaptation reactor, resulting in the release of 11.9 mg trehalose/g VSS. Genome analysis shows the possibility of *Ca.* Accumulibacter phosphatis to convert glycogen into trehalose by the presence of *treX*, *treY*, and *treZ* genes. Addition of trehalose to the reactor led to its consumption, both during anaerobic and aerobic phases. These results indicate the flexibility of the metabolism of *Ca.* Accumulibacter phosphatis towards variations in salinity.

**Key points:**

• *Trehalose is identified as an osmolyte in Candidatus Accumulibacter phosphatis.*

• *Ca*. *Accumulibacter phosphatis can convert glycogen into trehalose.*

• *Ca*. *Accumulibacter phosphatis clade I is present and active in both seawater and freshwater.*

**Electronic supplementary material:**

The online version of this article (10.1007/s00253-020-10947-8) contains supplementary material, which is available to authorized users.

## Introduction

Enhanced biological phosphorus removal (EBPR) is an important technology for the removal of phosphate from wastewater streams (Blackall et al. 2002; Seviour et al. 2003; Singh and Srivastava 2011). Anaerobic uptake of volatile fatty acids (VFA) and aerobic uptake of phosphate by phosphate-accumulating organisms (PAO) lead to the complete removal of both chemical oxygen demand (COD) and phosphate in one process step (Comeau et al. 1986; Smolders et al. 1994). This process has been extensively studied under freshwater conditions. Saline process conditions have also been investigated, and recently, it has been shown that also seawater-based influents can lead to good EBPR efficiency (Wang et al. 2009; Guo et al. 2018; De Graaff et al. 2020). Most interestingly, short-term exposure of this seawater-adapted culture to freshwater led to the release of COD, which was proposed to be related to osmolyte excretion.

Intracellular accumulation of osmolytes is a common mechanism for halophilic organisms to survive in saline environments (da Costa et al. 1998; Roeßler and Müller 2001). Osmolytes, sometimes also called compatible solutes or osmoprotectants, are small organic molecules that can be either zwitterionic, noncharged, or anionic, which are used to balance internal and external osmotic pressure without interfering with cellular processes (Sleator and Hill 2001; Roberts 2005). There is a large range of osmolytes that are widely conserved among organisms ranging from plants to bacteria and fungi (Yancey et al. 1982; Csonka 1989; Klipp et al. 2005). Production of osmolytes can be induced by an increase in external osmotic pressure, and release is triggered by a decrease in osmotic pressure (Guillouet and Engasser 1995; Kempf and Bremer 1998; Sauer and Galinski 1998).

This behavior signifies the importance of understanding osmolyte metabolism in wastewater systems that might undergo dynamic changes in salt content of the influent, as can occur due to industrial discharges or seawater intrusion in the sewer system. The role of osmolytes in saline wastewater treatment has regularly been reported for nitrifying sludge and methanogenic sludge (Corsino et al. 2016; Wang et al. 2017; Vyrides and Stuckey 2017; Sudmalis et al. 2018). The most detailed salinity effect described for *Candidatus* Accumulibacter phosphatis is an increase in maintenance coefficients after adaptation to NaCl-based influent (Welles et al. 2014). Identification and production of osmolytes or their function within *Ca.* Accumulibacter phosphatis under saline conditions have not been described in literature.

In this paper, we identified and described the role of trehalose as an osmolyte in a *Ca.* Accumulibacter phosphatis enrichment culture. A freshwater-adapted enrichment culture was adapted to a salinity level of 30% seawater. The impact on EBPR efficiency and microbial community composition was analyzed. The enrichment cultures were exposed to hyperosmotic incubation followed by hypo-osmotic shock, to study the effect on trehalose production and release. The aerobic and anaerobic uptake of trehalose was studied. The link between glycogen storage pool and trehalose metabolism and the impact of this so-far unknown carbon pool on the EBPR processes are discussed.

## Materials and methods

### Reactor operation

A freshwater-adapted *Ca.* Accumulibacter phosphatis enrichment reactor was operated as described in Guedes da Silva et al. (2018). This reactor was subsequently adapted to a salinity level of 30% seawater with the following operational parameters.

A 1.5-L stirred tank reactor was operated as a sequencing batch reactor (SBR) with a 6-h cycle. Each cycle consisted of 20 min settling, 15 min effluent withdrawal, 5 min N_2_ sparging, 5 min feeding, 135 min anaerobic phase, and 180 min aerobic phase. The sludge retention time (SRT) was maintained at 10 days through a short cyclical effluent removal during the mixed aerobic phase. The hydraulic retention time (HRT) was equal to 12 h (50% exchange ratio). pH was controlled at 7.0 ± 0.1 by dosing either 0.4 M HCl or 0.4 M NaOH. Temperature was controlled at 20 ± 1 °C. Conductivity in the bulk liquid was used to follow phosphate release and uptake profiles as described in Kim et al. (2007). The on-line profiles were used to verify steady operation of the reactor.

The feed of 750 mL was combined from two separate media: a concentrated COD medium and a concentrated mineral medium, which were both diluted with artificial seawater (final concentration of 10 g/L Instant Ocean® sea salts) prior to feeding into the reactor. The combination of these solutions led to influent concentrations of 400 mg COD/L (260 mg COD/L from NaAc·3H_2_O and 140 mg COD/L from NaPr), 50 mg PO_4_^3−^-P/L (from 222 mg/L NaH_2_PO_4_·3H_2_O), 40 mg NH_4_^+^-N/L (from 152.5 mg/L NH_4_Cl), 158.6 mg/L MgSO_4_·7H_2_O, 48 mg/L KCl, 40 mg/L CaCl_2_·2H_2_O, 4 mg/L N-allylthiourea, 4 mg/L yeast extract, and 0.6 mL/L trace element solution (Vishniac and Santer 1957).

Trehalose uptake experiments in the reactor were performed by addition of a stock solution (5 mL) of trehalose that was prepared in artificial seawater (10 g/L Instant Ocean® sea salts, similar to the reactor concentration). This solution was manually added to the reactor at either the start of aeration or the start of the anaerobic phase. The final concentration of trehalose in the reactor was 100 mg/L. When this trehalose solution was added at the start of the anaerobic phase, acetate and propionate were not added to the reactor.

### Batch tests

Sludge was taken from the reactor at the end of the aerobic reactor cycle, and transferred to flasks with 100 mL working volume. These flasks contained either demineralized water (buffered at pH 7.0 ± 0.1 with 4.0 mM HEPES buffer) or a solution of Instant Ocean® sea salts, and were sparged with nitrogen gas prior to adding the sludge. Samples were taken over time and filtered through a 0.45-μm PVDF filter. After the anaerobic phase, the sludge was allowed to settle in the flask, and liquid was decanted and replaced by 100 mL demineralized water, and aerated for a duration of 60 min. No nutrients were added to the liquid during these batch tests.

The respective masses of all samples were registered to compensate for mass decrease during calculations. The amount of biomass was determined by filtering the granules at the end of the test, washing with demineralized water to remove salts, drying for 24 h at 105 °C, and burning for 2 h at 550 °C. All tests were done in duplicate.

### Analytical methods

Concentrations of phosphate were measured on a Thermo Fisher Gallery Discrete Analyzer (Thermo Fisher Scientific, Waltham, USA). Acetate and propionate were measured by HPLC with an Aminex HPX-87H column from Biorad, coupled to an RI and UV detector, using 0.01 M phosphoric acid as eluent. Trehalose analysis was performed with a Dionex ICS-5000^+^ (Thermo Fisher Scientific, USA), a CarboPac PA20 3*150 mm column, and an Amino Trap 3*30 mm pre-column (column temperature 30 °C). Injection volume was 2–10 μL. Eluents used were ultrapure water, 200 mM NaOH, and 50 mM sodium acetate with 200 mM NaOH. Flow equaled 0.5 mL/min, isocratic with 10 mM NaOH during 20 min, followed by a cleaning step with 200 mM NaOH for 20 min and sodium acetate for 60 min in total. Samples were analyzed pulsed amperometrically with a quadruple wave form, with gold electrode and Ag/AgCl reference electrode.

### Fluorescence in situ hybridization

The handling, fixation, and staining of fluorescence in situ hybridization (FISH) samples were performed as described in Bassin et al. (2011). A mixture of PAO462, PAO651, and PAO846 probes (PAOmix) was used for visualizing polyphosphate-accumulating organisms (PAO) (Crocetti et al. 2000). A mixture of GAOQ431 and GAOQ989 probes (GAOmix) was used for visualizing glycogen-accumulating organisms (GAO) (Crocetti et al. 2002). A mixture of EUB338, EUB338-II, and EUB338-III probes was used for staining all bacteria (Amann et al. 1990; Daims et al. 1999). *Ca.* Accumulibacter clade I was visualized by Acc-I-444, and *Ca.* Accumulibacter clade II was visualized by Acc-II-444 (Flowers et al. 2009). Images were taken with a Zeiss Axioplan 2 epifluorescence microscope equipped with filter set 26 (bp 575e625/FT645/bp 660e710), 20 (bp 546/12/FT560/bp 575e640), and 17 (bp 485/20/FT 510/bp 5515e565) for Cy5, Cy3, and fluos, respectively.

The biovolume of PAO was calculated by counting the amounts of colored pixels from the FISH images, calculated with simple in-house software: The amount of pixels that were colored with the EUB338 probe (staining all eubacteria) was used as 100%, and the amount of pixels that were colored with the PAOmix probes (PAO462, PAO651, and PAO846 probes) was used for calculation of their respective fraction. A total of 10 representative pictures were used for calculation of the PAO fraction.

### Amplicon sequencing

DNA was extracted using the DNeasy UltraClean Microbial Kit (Qiagen, The Netherlands). Approximately 250 mg wet biomass was treated according to the standard protocol except an alternative lysis was implemented. This included a combination of 5 min of heat (65 °C) followed by 5 min of bead-beating for cell disruption on a Mini-Beadbeater-24 (Biospec, USA). After extraction, the DNA was checked for quality by gel electrophoresis and quantified using a Qubit 4 (Thermo Fisher Scientific, USA).

After quality control, samples were sent to Novogene Ltd. (Hong Kong, China) for Amplicon sequencing of the V3–4 region of the 16S-rRNA gene (position 341–806) on an Illumina paired-end platform. After sequencing, the raw reads were quality filtered, chimeric sequences were removed, and operational taxonomic units (OTUs) were generated on the base of ≥ 97% identity. Subsequently, microbial community analysis was performed by Novogene using Mothur & Qiime software (V1.7.0). For phylogenetical determination, a most recent SSURef database from SILVA (http://www.arb-silva.de/) was used.

### Genome analysis

Fifty-nine available metagenome sequences of *Candidatus* Accumulibacter phosphatis enrichment cultures were obtained from JGI IMG database. These metagenomes were compared with protein sequences of TreX, TreY, and TreZ proteins, obtained from the NCBI protein database. BLASTp was performed using the on-line BLASTp tool by JGI IMG. Alignment was performed according to the algorithm as described in Altschul et al. (1997) and Schäffer et al. (2001). Lower *E*-values indicate a lower uncertainty in the presence of certain sequences. Values lower than 1E−40 were set as threshold for positive results.

## Results

### Trehalose release from freshwater-adapted *Ca.* Accumulibacter phosphatis enrichment culture

The freshwater-adapted enrichment culture of *Ca.* Accumulibacter phosphatis was operated as described in Guedes da Silva et al. (2018). This reactor had a *Ca.* Accumulibacter phosphatis fraction of > 90% of the biovolume as determined by fluorescence in situ hybridization (FISH) analysis. No trehalose was measured in the reactor during a normal cycle of anaerobic COD uptake and phosphate release, and aerobic phosphate uptake.

**Fig. 1:**
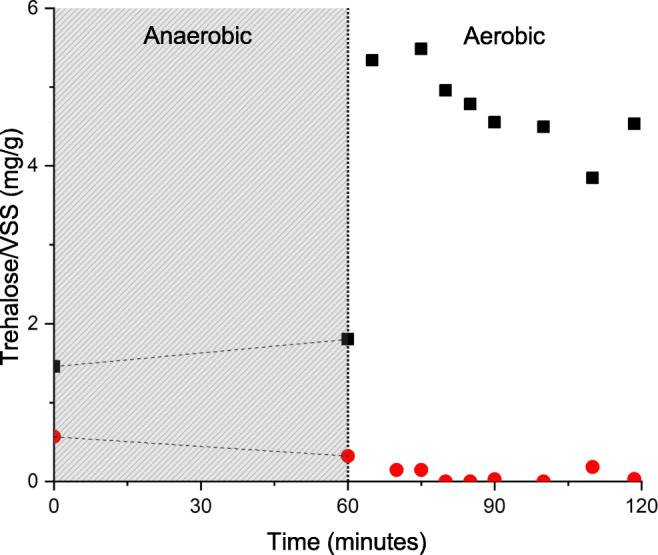
Trehalose release of freshwater-adapted *Ca.* Accumulibacter phosphatis enrichment culture after 60 min anaerobic incubation in either 30% seawater (black squares) or demineralized water (red circles), followed by exposure to aerated demineralized water. The gray area denotes the anaerobic period, and the white area notes the aerobic period

This freshwater-adapted sludge was used in a batch test to assess the potential for intracellular trehalose production. A sludge sample was anaerobically incubated in 10 g/L artificial seawater (30% of seawater) for 60 min without the presence of a COD source. Afterwards, the sludge was transferred to aerated demineralized water for another 60 min. No nutrients were added to assess the effect of only the salinity increase. A control sample was tested in the same way, but with anaerobic incubation in demineralized water instead of artificial seawater. Trehalose concentrations were measured in the liquid phase over time (Fig. 1). The anaerobic incubation of freshwater-adapted *Ca.* Accumulibacter phosphatis for 60 min in 10 g/L artificial seawater and subsequent transfer to a freshwater medium resulted in the release of 5.34 mg trehalose/g volatile suspended solids (VSS) within only 10 min after the osmotic downshock. The control experiment with anaerobic incubation in demineralized water yielded negligible release of trehalose after osmotic downshock.

These results signify the potential for freshwater-adapted *Ca.* Accumulibacter phosphatis sludge to adapt to a short-term sudden increase in salinity by producing trehalose. The next question is whether this enrichment culture can adapt to prolonged exposure to increased seawater salinity and how this influences trehalose production.

### Long-term adaptation of *Ca.* Accumulibacter phosphatis to seawater

A *Ca.* Accumulibacter phosphatis enrichment reactor was operated with 30% seawater (10 g/L total dissolved salts). Acetate and propionate were added anaerobically and completely consumed within 30 min after feeding. Phosphate was released up to 167 mg PO_4_^3−^-P/L (0.73 Pmol/Cmol), and completely taken up during aeration. Stable on-line measurements of pO_2_, pH, and conductivity confirmed the occurrence of a pseudo steady state during 126 days of stable operation.

### Microbial community analysis

**Fig. 2:**
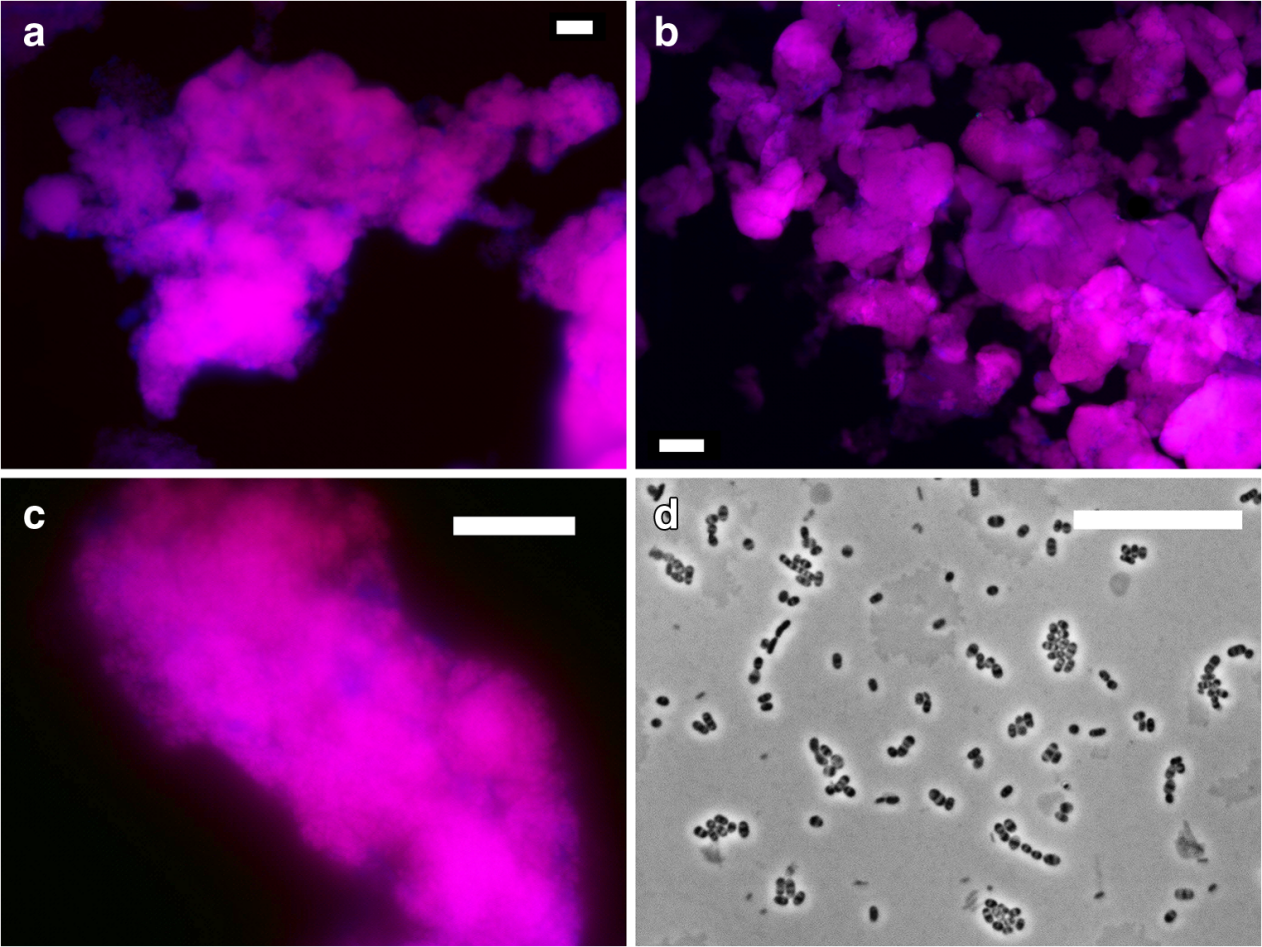
Fluorescence in situ hybridization (FISH) analysis of 30% seawater-adapted *Ca.* Accumulibacter phosphatis enrichment culture, with PAOmix (cy3, red; **a**), GAOmix (fluos, green), EUB338 (cy5, blue); **b**, **c**), *Ca.* Accumulibacter clade I (cy3, red), *Ca.* Accumulibacter clade II (fluos, green), EUB338 (cy5, blue); **d** brightfield microscopy image showing *Ca.* Accumulibacter clade I morphology. Scale bar equals **a** 20 μm or **b** 100 μm

The microbial community of the seawater-adapted culture was analyzed by means of fluorescence in situ hybridization (FISH) and next-generation amplicon sequencing (NGS). FISH analysis with PAO-specific probes and GAO-specific probes showed abundance of PAO and complete absence of GAO (Fig. 2a). The biovolume of the 30% seawater-adapted PAO community as deduced from the FISH microscopy was > 90%. Clade differentiation by means of *Ca.* Accumulibacter clade I- and clade II-specific probes showed the presence of clade I, while clade II was not observed (Fig. 2b, c). The typical morphology of *Ca.* Accumulibacter clade I was verified with brightfield microscopy imaging (Fig. 2d).

**Fig. 3:**
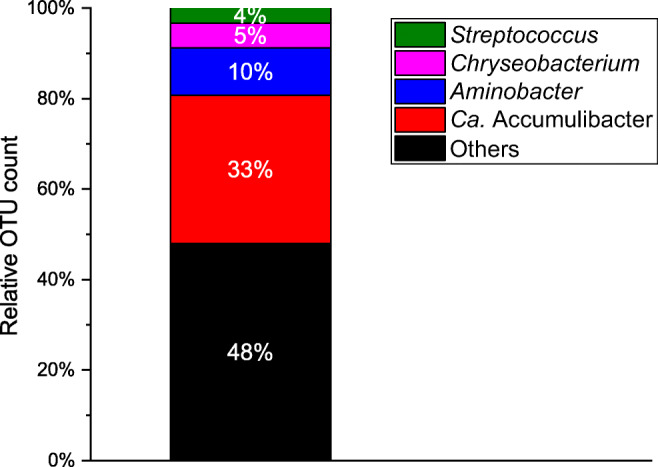
Relative OTU count of the most abundant genera of a 30% seawater-adapted *Ca.* Accumulibacter phosphatis enrichment culture

NGS analysis showed only a 33% relative abundance of *Ca.* Accumulibacter (Fig. 3). The relative OTU count of *Ca.* Accumulibacter is much lower than the fraction that was observed with FISH microscopy, which could be due to bias in extraction and quantification of PAO cultures (Stokholm-Bjerregaard et al. 2017).

### Trehalose release from seawater-adapted *Ca.* Accumulibacter phosphatis sludge

**Fig. 4:**
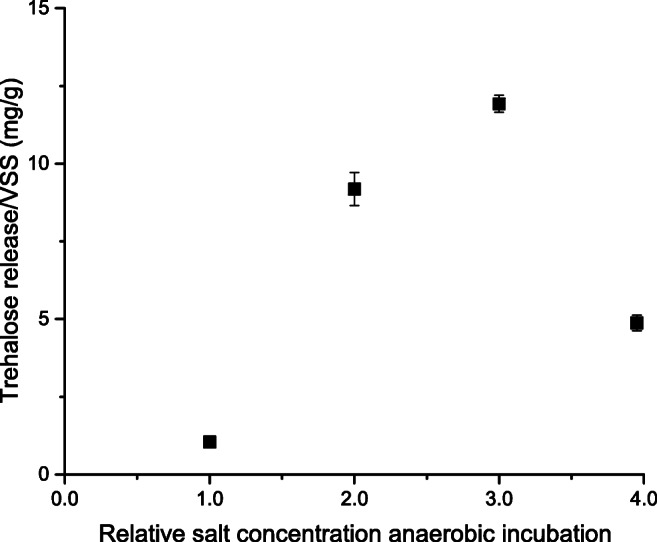
Concentrations of released trehalose by the 30% seawater-adapted *Ca.* Accumulibacter phosphatis enrichment culture, during aerated downshock in demineralized water, after 60 min anaerobic incubation in either 1×, 2×, 3×, or 4× relative salt concentration compared to 30% seawater

A single cycle of hyperosmotic incubation and hypo-osmotic shock in a freshwater-adapted *Ca.* Accumulibacter phosphatis enrichment culture caused the release of 5.34 mg trehalose/g VSS (Fig. 1). This led to the question whether seawater-adapted *Ca.* Accumulibacter phosphatis will release more trehalose, and whether exposure to even higher osmotic pressures will also lead to more intracellular trehalose production. Therefore, the seawater-adapted enrichment culture was anaerobically incubated in different concentrations of salinity (hyperosmotic incubation). Subsequently, they were exposed to demineralized water in order to release their produced trehalose (hypo-osmotic shock), similar to the experiment in Fig. 1. Trehalose was measured in the liquid phase during the aerated downshock (Fig. 4).

The control sample of 30% seawater (1× relative salinity) led to the release of 1.0 mg trehalose/g VSS after hypo-osmotic shock in demineralized water, within 5 min of the hypo-osmotic shock. This amount increased to 9.2 mg trehalose/g VSS and 11.9 mg trehalose/g VSS after anaerobic incubation in 2× and 3× relative salinity, respectively. After anaerobic incubation in four times relative salinity, the amount of released trehalose decreased to 4.9 mg trehalose/g VSS. Negligible amounts of trehalose uptake over time were measured during 60 min of aeration. The amount of phosphate release was similar in all samples (between 8.4 and 9.1 mg/L within 10 min of hypo-osmotic shock).

### Trehalose uptake by seawater-adapted *Ca.* Accumulibacter phosphatis enrichment culture

**Fig. 5:**
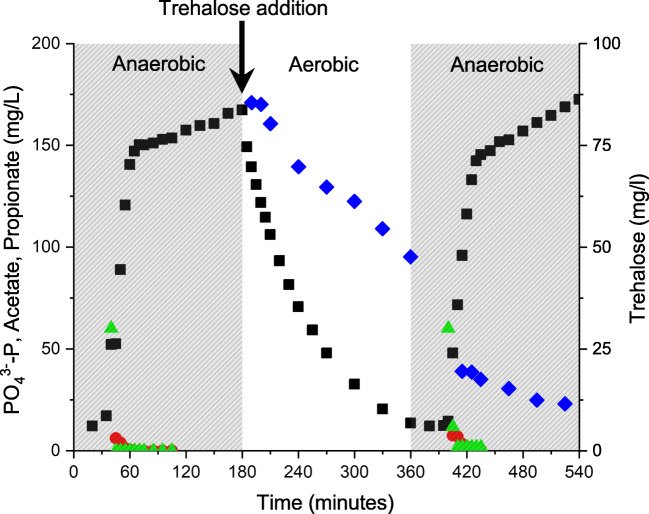
Aerobic addition of trehalose in the 30% seawater-adapted *Ca.* Accumulibacter phosphatis enrichment reactor. Concentrations of phosphate (black squares), acetate (red circles), propionate (green triangles), and trehalose (blue diamonds) are measured over time. The gray areas denote the anaerobic periods, and the white area denotes the aerobic period

**Fig. 6:**
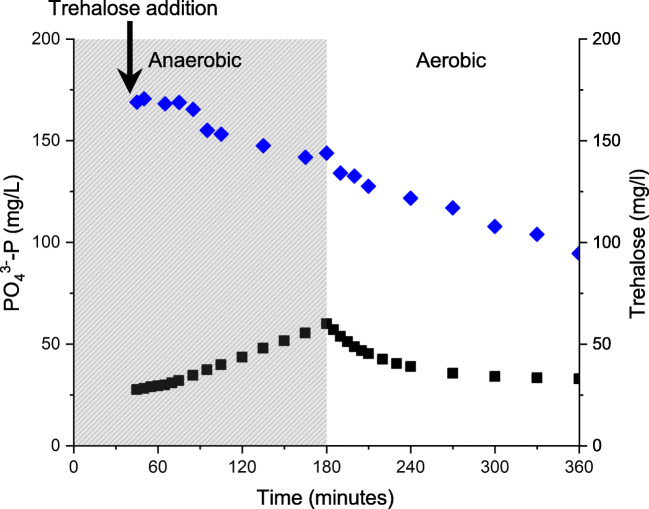
Anaerobic addition of trehalose by replacement of acetate and propionate during feeding in the 30% seawater-adapted *Ca.* Accumulibacter phosphatis enrichment reactor. Concentrations of phosphate (black squares) and trehalose (blue diamonds) are measured over time. The gray-out area denotes the anaerobic period, and the white area notes the aerobic period

Our previous results showed the capacity of intracellular trehalose production, but uptake of external trehalose can give great insight into a potential new carbon source for culturing *Ca.* Accumulibacter phosphatis. Therefore, trehalose was added to the reactor during both aeration (Fig. 5) and anaerobic feeding, replacing acetate and propionate as the COD source (Fig. 6). Characteristic values are shown in Table 1.

**Table 1:** Characteristic values of the 30% seawater-adapted *Ca.* Accumulibacter phosphatis enrichment culture after addition of trehalose to the enrichment reactor

	Regular cycle	Aerobic trehalose addition	Subsequent anaerobic cycle	Anaerobic trehalose addition	Subsequent aerobic cycle
Trehalose present	**–**	**+**	**+**	**+**	**+**
Acetate + propionate present	**+**	**–**	**+**	**–**	**–**
Aerobic trehalose uptake rate (mg trehalose/g VSS/h)		8.0			11.2
Anaerobic trehalose uptake rate (mg trehalose/g VSS/h)			2.8	9.7	
Maximal aerobic P-uptake rate (mg P/g VSS/h)	53.3	47.8			18.5
Maximal anaerobic P-release rate (mg P/g VSS/h)	262.0		208.8	9.5	
Anaerobic P-release after COD uptake (mg P/g VSS/h)	6.3		9.3		
Net anaerobic phosphate release (mg PO_4_^3−^-P/L)	161.2		166.4	56.9	

Values are shown for both the aerobic and anaerobic phases during which trehalose was added, and for the subsequent anaerobic or aerobic phase, respectively

Trehalose was taken up both aerobically and anaerobically. After aerobic addition, phosphorus uptake continued with a similar rate compared to the regular phosphorus uptake rate (without the presence of any trehalose). Not all trehalose was taken up aerobically, so it leaked into the subsequent anaerobic cycle during which fresh VFA (acetate and propionate) were added to the reactor sludge. The presence of trehalose did not interfere with VFA uptake, but the secondary phosphorus release rate after VFA uptake increased from 6.3 to 9.3 mg P/g VSS/h. Complete replacement of VFA by trehalose in the anaerobic influent gave a similar phosphorus release rate of 9.5 mg P/g VSS/h. The aerobic phosphorus uptake rate decreased by 65% compared to the regular reactor cycle with anaerobic VFA consumption.

### Metabolic pathway analysis for trehalose production and uptake in *Ca.* Accumulibacter phosphatis

An increase in osmotic pressure led to an increase in trehalose production (Fig. 1, Fig. 4). This raises the question through which metabolic pathway this production occurs. Conversion of glycogen into trehalose is a commonly described pathway (Elbein et al. 2003; Pade et al. 2014). The presence of glycogen pools in *Ca.* Accumulibacter phosphatis makes this pathway a suitable candidate for analysis.

Conversion of glycogen into trehalose commonly occurs in a two-step TreYZ pathway (Maruta et al. 1996; Ruhal et al. 2013). TreY converts the α-1,4 bonds in glycogen into α-1,1 bonds, from which trehalose disaccharides are cleaved by TreZ. This set of reactions can be aided by the presence of TreX, which is capable of debranching glycogen chains (Seibold and Eikmanns 2007).

Uptake of trehalose by bacteria commonly involves breakdown of trehalose into glucose monomers by trehalase (Boos et al. 1990). In *Escherichia coli*, for example, trehalose is broken down into glucose monomers by periplasmic trehalase, and subsequently taken up by the phosphotransferase system as glucose-6-phosphate (Gutierrez et al. 1989). Presence of the *treA* gene that encodes for trehalase can indicate whether *Ca.* Accumulibacter phosphatis has a similar mechanism.

A total of 59 *Ca.* Accumulibacter phosphatis metagenomes have been analyzed for the presence of genes that encode for TreY, TreZ, TreX, and TreA (Supplemental Material 1). Protein sequences have been used from other bacterial species, and these have been aligned to the metagenome of *Ca.* Accumulibacter phosphatis cultures. Lower *E*-values indicate a higher certainty of the gene being present in the genome. All genes that encode for TreY, TreZ, TreX, and TreA have very low *E*-values, close to or equal to zero. This indicates that the probability for conversion of glycogen to trehalose through the TreYZ pathway is highly likely. Uptake of trehalose can similarly occur by hydrolysis of trehalose into glucose monomers.

## Discussion

### Trehalose in *Candidatus* Accumulibacter phosphatis

This study shows the presence and role of trehalose in *Ca.* Accumulibacter phosphatis. Excretion of trehalose after a hypo-osmotic shock revealed the presence of intracellular trehalose pools. Both freshwater-adapted and seawater-adapted cultures produce trehalose after a hyperosmotic incubation, which indicates the remarkable flexibility of *Ca.* Accumulibacter phosphatis towards salinity variations. These results signify the importance of trehalose in salt adaptation, and can explain the flexibility of the EBPR process towards increased salinity (De Graaff et al. 2020).

The presence of trehalose is of major importance for accurately describing the metabolism of *Ca.* Accumulibacter phosphatis. Metabolic models have so far not included the presence of this compound. Quantification of intracellular trehalose pools will be essential for further research. However, common methods for glycogen quantification by extraction and analysis of its hydrolyzed glucose monomers could be interfering, since trehalose is also composed of glucose monomers (Elbein and Mitchell 1973). Previous studies could potentially even have overestimated their glycogen pools, if trehalose would have been present in significant amounts.

### Metabolic link between glycogen and trehalose

A metabolic link between glycogen and trehalose in *Candidatus* Accumulibacter phosphatis is proposed. This link is similar to *Saccharomyces cerevisiae*, where glycogen and trehalose have commonly been described in conjunction (François and Parrou 2001). Trehalose can be produced from glycogen through a simple 2-step enzymatic reaction by TreY and TreZ enzymes (Maruta et al. 1996). TreY can convert α(1–4) glucose polymers such as glycogen into maltooligosyl-trehalose, while TreZ can subsequently hydrolyze into trehalose sugars.

Genome analysis shows that *treX*, *treY*, and *treZ* genes are present in the genome of *Ca.* Accumulibacter phosphatis. The results presented here indicate that trehalose can be produced very rapidly (Fig. 1, Fig. 4), likely enabled by these enzymes. A deeper understanding of the exact regulation of conversion of glycogen into trehalose will lead to improvement of metabolic models. Stoichiometric and kinetic quantification of intracellular metabolite concentrations will be key for filling the gaps in this pathway.

Excreted trehalose is also taken up again albeit at a relatively low rate. *Ca.* Accumulibacter phosphatis is described as not using glucose, but trehalose seems to be a sugar substrate that is metabolized by this species. This can occur through hydrolysis of trehalose by a trehalase enzyme and subsequent use of the produced glucose. There is, however, also a direct pathway described for conversion of trehalose into glycogen, but this seems limited to mycobacteria (Chandra et al. 2011).

### Optimum in trehalose release after short-term incubation in higher salinities

Hyperosmotic incubation has frequently been described to be a trigger for intracellular osmolyte production (Guillouet and Engasser 1995; Skjerdal et al. 1996; Kempf and Bremer 1998; Morbach and Krämer 2003). The amount of trehalose released after hypo-osmotic shock increased with the salinity level during the preceding anaerobic hyperosmotic incubation. Trehalose release per VSS increased from 2.1 mg trehalose/g VSS at the adapted level of 30% seawater, to 12 mg trehalose/g VSS at three times this salinity. Incubation in four times the adapted salinity gives a much lower release of 4.9 mg trehalose/g VSS (Fig. 4).

A common response to hyperosmotic incubation is the downregulation of the metabolic rate in both eukaryotic and prokaryotic cells (Bishop et al. 1994; Dai and Zhu 2018). Shrinking of bacterial cells leads to an increase in metabolite and protein concentrations, also known as molecular crowding (Zimmerman and Minton 1993). This can result in a decrease in diffusion of proteins and a change in occurrence of metabolic reactions (Lang et al. 1998; Ellis and Minton 2003; Dorsaz et al. 2010; Miermont et al. 2013).

Intracellular conversion of glycogen into trehalose can therefore have a lower rate at higher salinity. The 1-h hyperosmotic incubation could have been too short to reach maximal trehalose concentrations. Longer-term salt adaptation would still be a viable option, due to both the longer time for trehalose production and the decrease in macromolecular crowing after longer duration of hyperosmosis (Liu et al. 2019).

### Clade I present in both freshwater-adapted and seawater-adapted *Ca.* Accumulibacter phosphatis sludge

*Ca.* Accumulibacter phosphatis clade I was the dominant clade in cultures that were adapted to freshwater and cultures that were adapted to 30% seawater (Fig. 2). This flexibility of a single organism to thrive in different levels of salinity is remarkable, especially compared to other commonly studied bacteria in wastewater treatment such as nitrifying bacteria and Anammox bacteria (Moussa et al. 2006; Kartal et al. 2006; Bassin et al. 2011; Borin et al. 2013; Gonzalez-Silva et al. 2016).

The adaptability of *Ca.* Accumulibacter phosphatis to a range of salinities is in line with the estuarine environments in which they were found in nature. This habitat is prone to cyclical variations in salinity, aerobicity, and nutrient availability, due to tidal fluctuations and mixing of seawater and freshwater (Pritchard 1952; Boynton and Kemp 1985). *Ca.* Accumulibacter has been found in the sediment-water interface of these dynamic environments (Davelaar 1993; Hupfer et al. 2007; Watson et al. 2019). Interestingly, clade I has been observed more frequently than clade II (Peterson et al. 2008). The newfound presence of trehalose as an osmolyte can add to the understanding of the adaptation strategy of *Ca.* Accumulibacter to these environments.

## Electronic supplementary material

ESM 1 (DOCX 16.8 kb)

## Data Availability

Data is available upon request.
