# Supplementary material for: Trehalose as an osmolyte in Candidatus Accumulibacter phosphatis
Source: Appl Microbiol Biotechnol. 2020 Oct 19;105(1):379–88. doi: 10.1007/s00253-020-10947-8 (PMC7778627; doi:10.1007/s00253-020-10947-8)
Supplement: Supplementary file 1 — (DOCX 16.8 kb) [file 253_2020_10947_MOESM1_ESM.docx]

Supplementary Material 1

**Table S1**. Genome analysis of enzymes that are involved in conversion of glycogen to trehalose (TreY, TreZ, TreX), and involved in the hydrolysis of trehalose for import (TreA).

| Enzyme  Accession number  Reference species | E-value | Metagenome name^*^ |
| --- | --- | --- |
| TreY | 1.00E-173 | a |
| CCE37077.1 | 3.00E-163 | a |
| *Mycobacterium tuberculosis* | 7.00E-156 | b |
|  | 6.00E-156 | c |
|  | 7.00E-156 | d |
| TreY | 0 | a |
| ABV26725.1 | 0 | a |
| *Actinoplanes sp.* | 0 | e |
|  | 3.00E-179 | c |
|  | 2.00E-179 | d |
| TreZ | 0 | a |
| CCE37076.1 | 0 | a |
| *Mycobacterium tuberculosis* | 0 | f |
|  | 0 | g |
|  | 0 | h |
| TreZ | 0 | g |
| ABV26726.1 | 0 | h |
| *Actinoplanes sp.* | 0 | i |
|  | 0 | j |
|  | 0 | k |
| TreX | 0 | f |
| CCE37078.1 | 0 | a |
| *Mycobacterium tuberculosis* | 0 | k |
|  | 0 | h |
|  | 0 | i |
| TreX | 0 | a |
| ABV26724.1 | 0 | a |
| *Actinoplanes sp.* | 0 | l |
|  | 0 | m |
|  | 0 | m |
| TreA | 8.00E-170 | n |
| EGT67795.1 | 2.00E-169 | o |
| *Escherichia coli* | 1.00E-169 | p |
|  | 3.00E-169 | q |
|  | 8.00E-170 | r |
| TreA | 0 | q |
| CDO15159.1 | 0 | n |
| *Klebsiella pneumoniae* | 0 | o |
|  | 0 | p |
|  | 0 | s |
|  |  |  |

1. Wastewater treatment Type I *Accumulibacter* community from EBPR Bioreactor in Madison, WI, USA - Reactor 2_5/28/2013_ DNA (Illumina Assembly)
2. Wastewater treatment Type I *Accumulibacter* community from EBPR Bioreactor in Madison, WI, USA - Reactor 1_1/23/2012_ DNA
3. Wastewater treatment Type I *Accumulibacter* community from EBPR Bioreactor in Madison, WI, USA - Reactor 2_5/13/2013_ DNA
4. Wastewater treatment Type I *Accumulibacter* community from EBPR Bioreactor in Madison, WI, USA - Reactor 1_10/4/2010_ DNA
5. Wastewater treatment Type I *Accumulibacter* community from EBPR Bioreactor in Madison, WI, USA - Reactor 1_2/2/2009_ DNA (SPAdes)
6. Wastewater treatment Type I *Accumulibacter* community from EBPR Bioreactor in Madison, WI, USA - Reactor 1_1/10/2011_ DNA
7. Wastewater treatment Type I *Accumulibacter* community from EBPR Bioreactor in Madison, WI, USA - Reactor 1_7/15/2010_ DNA
8. Wastewater treatment Type I *Accumulibacter* community from EBPR Bioreactor in Madison, WI, USA - Reactor 1_2/2/2009_ DNA
9. Wastewater treatment Type I *Accumulibacter* community from EBPR Bioreactor in Madison, WI, USA - Reactor 1_7/15/2010_ DNA (SPAdes)
10. Wastewater treatment Type I *Accumulibacter* community from EBPR Bioreactor in Madison, WI, USA - Reactor 1_4/24/2008_ DNA (SPAdes)
11. Wastewater treatment Type I *Accumulibacter* community from EBPR Bioreactor in Madison, WI, USA - Reactor 1_1/10/2011_ DNA (SPAdes)
12. Wastewater treatment Type I *Accumulibacter* community from EBPR Bioreactor in Madison, WI, USA - Reactor 2_5/28/2013_ DNA (Hybrid Assembly)
13. Wastewater treatment Type I *Accumulibacter* community from EBPR Bioreactor in Madison, WI, USA - Reactor 2_5/28/2013_ DNA (PacBio error correction)
14. Wastewater treatment Type I *Accumulibacter* community from EBPR Bioreactor in Madison, WI, USA - TNR Reactor, Time C-32min-Anaerobic_ RNA (Metagenome Metatranscriptome)
15. Wastewater treatment Type I *Accumulibacter* community from EBPR Bioreactor in Madison, WI, USA - TNR Reactor, Time B -10min-Anaerobic_ RNA (Metagenome Metatranscriptome)
16. Wastewater treatment Type I *Accumulibacter* community from EBPR Bioreactor in Madison, WI, USA - TNR Reactor_6/25/2014_ DNA
17. Wastewater treatment Type I *Accumulibacter* community from EBPR Bioreactor in Madison, WI, USA - TNR Reactor, Time F- 52min-Aerobic_ RNA (Metagenome Metatranscriptome)
18. Wastewater treatment Type I *Accumulibacter* community from EBPR Bioreactor in Madison, WI, USA - TNR Reactor, Time I- 292min-Aaerobic_ RNA (Metagenome Metatranscriptome)
19. Wastewater treatment Type I *Accumulibacter* community from EBPR Bioreactor in Madison, WI, USA - TNR Reactor, Time E -22min-Aerobic_ RNA (Metagenome Metatranscriptome)
